# Salivary Gland Tissue Engineering Approaches: State of the Art and Future Directions

**DOI:** 10.3390/cells10071723

**Published:** 2021-07-08

**Authors:** Lindsay R. Piraino, Danielle S. W. Benoit, Lisa A. DeLouise

**Affiliations:** 1 Department of Biomedical Engineering, University of Rochester, Rochester, NY 14627, USA; lpirain2@ur.rochester.edu (L.R.P.); danielle.benoit@rochester.edu (D.S.W.B.); 2 Center for Oral Biology, University of Rochester Medical Center, Rochester, NY 14642, USA; 3 Department of Biomedical Genetics, University of Rochester Medical Center, Rochester, NY 14642, USA; 4 Department of Environmental Medicine, University of Rochester Medical Center, Rochester, NY 14642, USA; 5 Wilmot Cancer Institute, University of Rochester Medical Center, Rochester, NY 14642, USA; 6 Materials Science Program, University of Rochester, Rochester, NY 14627, USA; 7 Department of Chemical Engineering, University of Rochester, Rochester, NY 14627, USA; 8 Center for Musculoskeletal Research, University of Rochester Medical Center, Rochester, NY 14642, USA; 9 Department of Dermatology, University of Rochester Medical Center, Rochester, NY 14642, USA

**Keywords:** salivary gland, tissue engineering, cell culture, soluble cues, media optimization

## Abstract

Salivary gland regeneration is important for developing treatments for radiation-induced xerostomia, Sjögren’s syndrome, and other conditions that cause dry mouth. Culture conditions adopted from tissue engineering strategies have been used to recapitulate gland structure and function to study and regenerate the salivary glands. The purpose of this review is to highlight current trends in the field, with an emphasis on soluble factors that have been shown to improve secretory function in vitro. A PubMed search was conducted to identify articles published in the last 10 years and articles were evaluated to identify the most promising approaches and areas for further research. Results showed increasing use of extracellular matrix mimetics, such as Matrigel^®^, collagen, and a variety of functionalized polymers. Soluble factors that provide supportive cues, including fibroblast growth factors (FGFs) and neurotrophic factors, as well as chemical inhibitors of Rho-associated kinase (ROCK), epidermal growth factor receptor (EGFR), and transforming growth factor β receptor (TGFβR) have shown increases in important markers including aquaporin 5 (Aqp5); muscle, intestine, and stomach expression 1 (Mist1); and keratin (K5). However, recapitulation of tissue function at in vivo levels is still elusive. A focus on identification of soluble factors, cells, and/or matrix cues tested in combination may further increase the maintenance of salivary gland secretory function in vitro. These approaches may also be amenable for translation in vivo to support successful regeneration of dysfunctional glands.

## 1. Introduction

**Figure 1:**
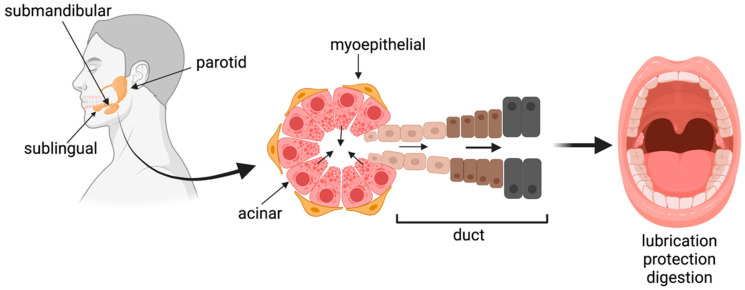
Schematic of the three major types of salivary glands, general gland structure, and major functions of saliva. Created with BioRender.com.

Salivary glands are organs that produce saliva, an aqueous fluid that contains enzymes, electrolytes, mucins, and other components that aid in lubricating the mouth, preliminary digestion, and antimicrobial and buffering activity to prevent oral infections [1]. There are three major pairs of salivary glands—the parotid, submandibular and sublingual—as well as numerous minor salivary glands located throughout the mouth [2]. The general gland structure consists of clusters of acinar cells, which secrete the proteins/fluid, and ductal cells, which modify the composition of the saliva and transport it into the oral cavity (Figure 1). There are also myoepithelial cells that surround the acinar units and other supportive tissues, including blood vessels, providing an exchange of nutrients and waste, and nerves, which play a key role in stimulating the glands to secrete [1]. More extensive reviews of salivary gland anatomy, physiology, and development can be found elsewhere [1,3,4,5].

The normal function of the salivary glands can be diminished by several conditions, such as off-target damage from radiation treatment for head or neck cancer, the autoimmune disease, Sjögren’s syndrome, some systemic conditions including diabetes and neurological diseases, and a vast array of medications, including anti-hypertensives, β-blockers, antidepressants, and many others [2,6,7]. The mechanisms underlying salivary gland dysfunction are not well understood and may vary greatly depending on the cause [6]. Therefore, existing treatments, including lubricating mouthwashes, gels, chewing gum, and sprays only ameliorate symptoms and provide temporary relief. Sialogogues that stimulate parasympathetic pathways, such as pilocarpine and cevimeline, have also been used, but success requires the patient to have some residual salivary function and they can cause systemic side effects [6]. Specific to radiation-induced salivary gland dysfunction, prevention is feasible by treating with amifostine, a reactive oxygen species scavenger, and/or utilizing intensity-modulated radiation therapy (IMRT), which is a targeted approach to avoid direct radiation to the parotid glands, but these methods come with drawbacks as well [6,8]. For example, amifostine can cause severe adverse side effects, including nausea, vomiting, allergic reactions, and hypotension, resulting in the need to discontinue treatment in up to 40% of cases [9]. Additionally, while IMRT has shown a benefit in preserving the saliva flow rate, patients may still suffer symptoms of xerostomia, possibly due to changes in saliva composition [10] and further, IMRT may not be possible based on tumor location [11]. Ultimately, incomplete understanding of the mechanism leading to salivary gland dysfunction has hindered the development of alternative treatment strategies and radioprotective drugs.

The use of in vitro culture systems would be useful to gain more knowledge on the mechanisms leading to salivary gland dysfunction as well as enable the development of new therapies, such as cell transplantation, the discovery of new radioprotective drugs, and methods to regenerate the damaged gland. However, culture of salivary glands has historically been difficult due to the rapid loss of secretory phenotype and function in vitro. For example, the characteristics of secretory acinar cells, such as mucin biosynthesis [12] and amylase production [13], decrease significantly after 1 day in culture. Expression of Mist1 (bHLH15a), a transcription factor necessary to specify the acinar cell phenotype [14], drops significantly as well [15,16]. Thus, tissue engineering approaches have been leveraged to address the requirement of functional tissue to enable drug discovery and screening, regeneration, and fundamental studies. In this review, we will discuss trends in salivary gland tissue engineering over the past decade with advantages/disadvantages of different culture methods. We identify areas of research that provide opportunities to improve in vitro culture of functional salivary gland tissue.

## 2. Materials and Methods

A literature review was conducted by searching for “salivary gland culture in vitro” in the PubMed database on 4 March 2021. The results were restricted to the past 10 years, giving a total of 320 articles. The results were screened to remove review articles and articles that did not contain in vitro culture, used nonmammalian cells, cultured only unhealthy tissue (ex. samples from a Sjögren’s syndrome or salivary gland cancer patient), or used non-salivary gland cell sources (ex. adipose, bone marrow, dental pulp) to differentiate into salivary gland-like structures. While these approaches are beneficial in expanding knowledge on the salivary gland, they likely demand vastly different culture conditions.

From the remaining 170 articles, various parameters were identified and categorized, as listed in Table 1. Categories included cell source, cell maturity, cell species, and gland type. Cell source was divided into *Primary* cells, *Cell line*, or *Both* while cell maturity indicated whether the study used *Adult*, *Embryonic*, or *Both* types of tissue; cell lines were considered under the *Adult* category. Cell species was split into *Human*, *Mouse*, *Rat*, *Other*, and *Multiple*; *Other* consisted of rarely used species (porcine, rabbit) and *Multiple* contained studies that used more than one cell species. Gland type was classified as *Submandibular*, *Parotid*, *Minor* (i.e., labial or other minor salivary glands), *Other*, or *Multiple*. *Other* consisted of sublingual or unspecified gland types while *Multiple* referred to studies that used more than one gland type.

Media type was divided into three groups based on the use of *Growth Factors*, such as epidermal growth factor (EGF) or fibroblast growth factor (FGF), the use of *Serum* including fetal bovine serum (FBS) and fetal calf serum (FCS), or *Commercial* media that contains proprietary or specialized additives optimized for a specific cell type. Overlap between these categories was considered. Individual media components were also extracted and listed separately.

Substrates were categorized into six groups: *Matrix mimetic*, *Plastic*, *Transfer*, *Specialized*, *Multiple*, *Membrane*, and *Unreported*. *Matrix mimetic* refers to substrates that are intended to mimic the function of the extracellular matrix (ECM) and was further divided into collagen, Matrigel^®^, and polymer sub-categories. *Plastic* indicates any kind of tissue culture polystyrene substrate, such as tissue culture plates, culture flasks, and Petri dishes. No distinction was made between tissue culture-treated and suspension plates, as this was often not reported in the literature. *Transfer* refers to cells that were cultured on one substrate and then transferred to another (ex. plastic for 7 days, then transferred to Matrigel^®^). *Specialized* includes commercially available platforms, such as hanging drop and GravityTRAP™ plates for 3D cell culture. The *Membrane* category contains substrates such as Transwells^®^ and Nuclepore™ polycarbonate filters. *Multiple* was used to classify articles that used multiple substrates or combined substrates from the other defined categories. *Unreported* was used to describe articles that did not clearly state the substrate used.

**Figure 2:**
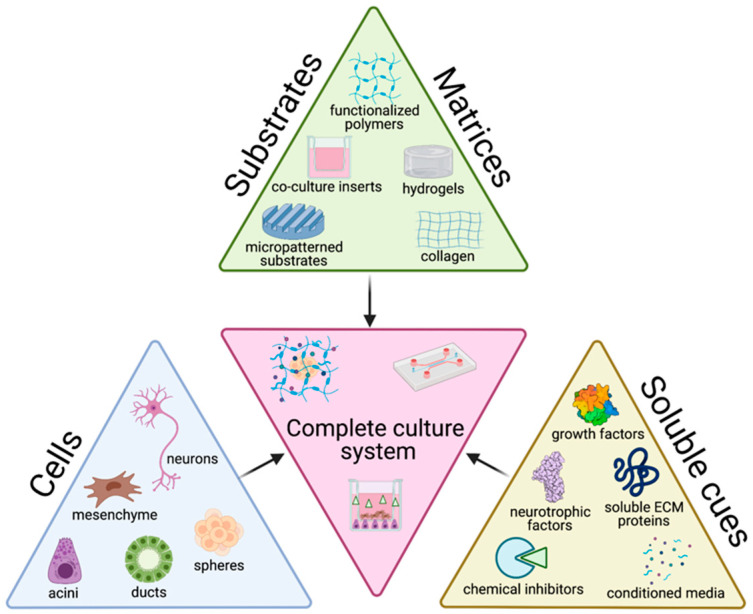
Schematic of the tissue engineering triad as it relates to salivary gland cell culture strategies with examples of a complete culture system.

Venn diagrams were created in JMP Pro 15.0.0 (SAS, Cary, NC, USA) using a Venn Diagram add-in. All other graphs were made in Prism (GraphPad, San Diego, CA, USA) version 8.4.3. Figure 1 and Figure 2 were created with BioRender.com (Science Suite, Inc., Toronto, ON, Canada).

## 3. Trends in In Vitro Salivary Gland Tissue Culture

The tissue engineering paradigm is typically comprised of three main components: cells, matrix, and soluble cues. Each of these components alone, as well as the interconnectedness between these three branches have important implications on the success of culture methods. To accurately recapitulate the structure and function of native tissue, the choice of cell source and the biophysicochemical cues provided to cells are key factors in determining the success of the culture with broader impacts on experimental significance. Here, we review the cells, matrices/substrates, and soluble factors and highlight some of the combinatory approaches that have utilized multiple categories, summarized in Figure 2. We also discuss the most beneficial approaches and identify opportunities for further research.

### 3.1. Cells

**Figure 3:**
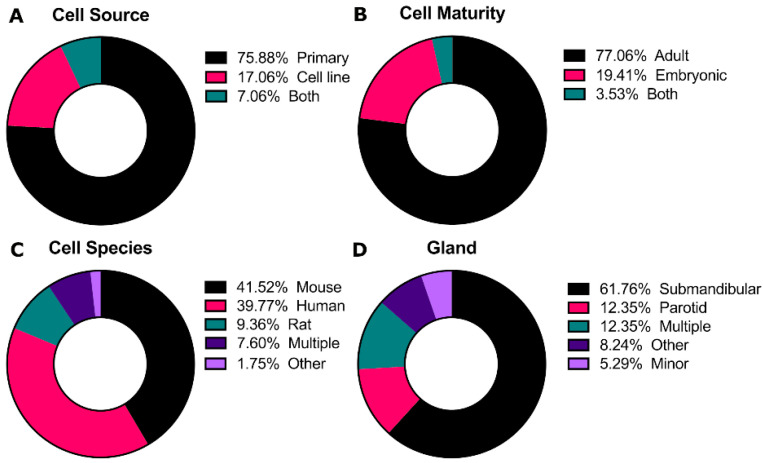
Categorization of the cells used in salivary gland culture in vitro. Distribution of the percentage frequency of the (**A**) cell source, **(B)** cell maturity, (**C**) cell species, and (**D**) gland type in the articles analyzed in this review.

Cells are the central aspect of the tissue engineering paradigm. For the salivary glands, cell lines do not accurately represent all characteristics of normal salivary gland tissue [17] and many of these cell lines are contaminated with HeLa cells, including the most commonly use HSG cell line [18,19]. Additionally, pluripotent stem cell technology for the salivary gland is in its infancy [20]. Thus, the majority of studies evaluated use primary cells for culturing salivary gland cells (76%, Figure 3A). Cells are most commonly sourced from adult tissue (77%, Figure 3B) of either mouse or human origins (42% and 40%, respectively, Figure 3C). The submandibular gland is the most frequently used (62%, Figure 3D), likely due to the relatively large cell yield and superficial location of the submandibular gland in mice [21] and the fact that the submandibular gland is the most well-studied in mice [15,16,22,23,24,25].

Primary cultures from adult tissue typically start with dissociation of the entire gland, containing acinar, duct, and myoepithelial cells. While some groups dissociate glands into single cells [23,26], others have highlighted the importance of maintenance of partial tissue structure to retain cell-cell contacts to promote 3D morphology and polarization [27,28,29,30]. For embryonic cultures, tissues are commonly grown as explants on a membrane with an air-liquid interface [31,32,33], although they can also be dissociated similar to adult tissue. In some cases, cells are selected for specific subpopulations using flow cytometry and/or selective enhancement during in vitro culture for putative stem cell markers such as CD24 and CD29, Kit, or K5 [34,35,36,37]. These studies typically aim to develop a cell transplantation strategy for regenerating the glands.

Co-cultures of salivary gland cells with other cell types, such as mesenchyme and nerves, have been investigated to support secretory function. For example, mouse cortical neurons were shown to self-organize around salivary gland cells similar to native tissue [38]. A crucial next step will be to determine if the neurons promote acinar characteristics in this model. Vining et al., 2019 showed that salispheres only undergo branching morphogenesis when combined with mesenchyme and parasympathetic ganglion in the presence of neurturin-containing matrices [23]. In addition, co-cultured endothelial cells were required for proper salivary gland epithelial patterning in embryonic explants [39]. These studies highlight the complexity of salivary gland tissue engineering and the need to consider multiple cell types. Further development in this area will be beneficial for creating a hierarchical tissue structure, as well as continued development of iPSC models.

### 3.2. Matrices/Substrates

The extracellular matrix (ECM) is a network of proteins, glycosaminoglycans, and proteoglycans that fills the intracellular space and provides structural and adhesive motifs that can influence a wide variety of cell functions such as proliferation, differentiation, and migration [40]. In the salivary gland epithelium, the ECM and basement membrane consist of laminin, collagen I, collagen IV, and fibronectin with binding sites for β1, β4, α5, and α6 integrins, among others [41]. These ECM proteins and their integrins orchestrate events during salivary gland development and homeostasis including branching morphogenesis, cleft formation, apico-basal polarization, adhesion, growth, and migration, and can influence intracellular signaling.

**Figure 4:**
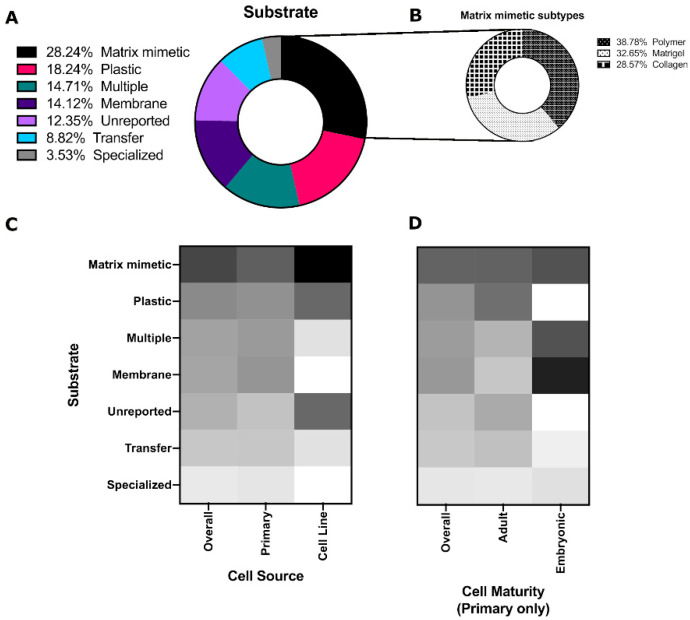
Categorization of the substrates used in salivary gland cell culture. Distribution of the percentage frequency of different (**A**) substrate categories and (**B**) substrates within the matrix mimetic category. Heat maps comparing the percentage frequency of different substrate categories by (**C**) cell source and (**D**) cell maturity for primary cells.

Given their importance in affecting cell behavior, it is not surprising that matrix mimetics were commonly used for salivary gland in vitro studies over the past decade (28%, Figure 4A). Of these, Matrigel^®^, collagen, and other polymers (mainly natural polymers) were equally as common (Figure 4B). Some of these matrices include: chitosan that increases salisphere size and number without increasing the size of the lumens, which are hollow openings within the spheroid [25]; hyaluronic acid-catechol that increases branching proliferation [42]; Matrigel^®^ that promotes 3D structure, amylase activity, tight junction formation and transepithelial resistance (TER) [43,44]; other laminin-containing matrices that can promote morphogenesis and Aqp5 expression in combination with FGF2 [22]; laminin hydrogels that induced branching morphogenesis and maintained epithelial progenitors [23]; matrix metalloproteinase (MMP)-degradable poly(ethylene glycol) (PEG) hydrogels that were found to promote polarized expression of zona occludins (ZO-1) and sodium potassium chloride channel 1 (Nkcc1) [15]; and human placenta basement membrane extract or fibronectin that were shown by transmission electron microscopy (TEM) to enhance tight junction formation [44].

Poly(styrene) substrates (tissue culture plates, Petri dishes, etc.) were the second most common culture platform, which were predominantly used for salivary gland cell lines (Figure 4C). Membranes (Nucleopore filters, Transwells) and multiple substrates (culturing on a Matrigel^®^ for some experiments and plastic for others) were more common for embryonic cells (Figure 4D). These polystyrene and coated substrates are standard, as many embryonic studies use explants and thus retain the structure of the developing gland without the need for extensive substrate engineering efforts.

Other substrates include: micropatterned PDMS-based craters with electrospun poly(lactic co glycolic acid) (PLGA) that were reported to increase the tight junction protein occuldin expression and expression of water channel Aqp5 [45]; poly(glycerol sebacate) (PGS)/PLGA core/shell nanofiber scaffolds that were reported to promote apical localization of tight junction proteins and tissue organization when combined with mesenchymal cells [46]; hanging drop cultures that were reported to produce microtissues mimicking tissue development [47]; and decellularized porcine gut matrix co-cultured with salivary gland cells and microvascular endothelial cells that were reported to promote amylase, claudin-1, and Aqp5 expression compared to 2D [48].

### 3.3. Soluble Cues

**Figure 5:**
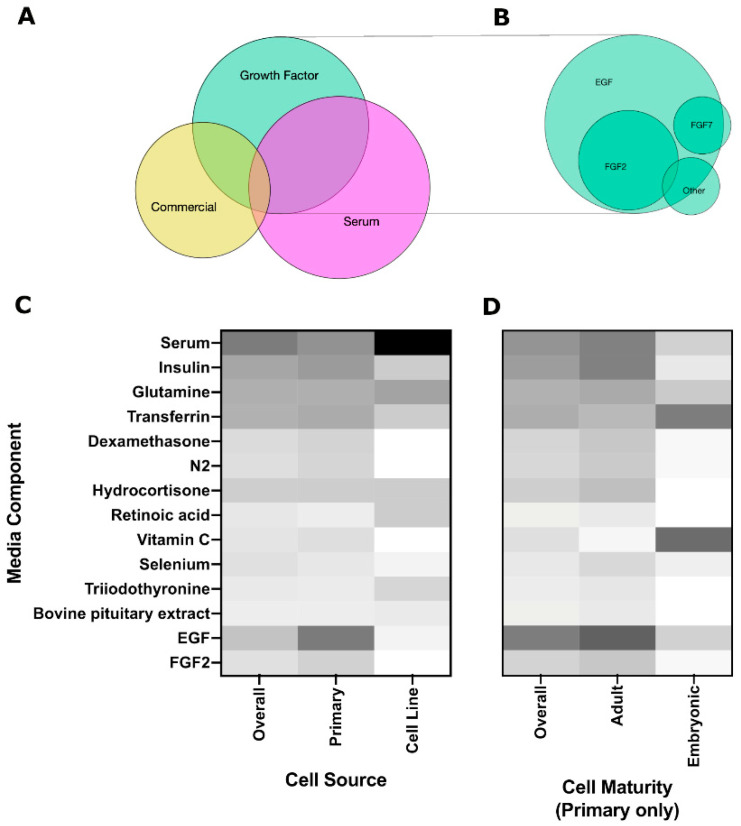
Categorization of the media type and percentage frequency of individual media components. Venn diagrams comparing the prevalence of different (**A**) media categories and (**B**) growth factors used in the growth factor-based media category. Heat maps comparing the percentage frequency of different media components by (**C**) cell source and (**D**) cell maturity for primary cells.

Soluble cues are typically provided to the cells through media supplementation. Salivary gland media commonly consists of Dulbecco’s Modified Eagle Medium (DMEM)/Ham’s F12 Nutrient Mixture with a variety of basic supplements to support cell growth in vitro, such as insulin, glutamine, transferrin, and antibiotics. Both serum-containing and growth factor-based media are common (Figure 5A), with EGF and FGF2 as the most common growth factors (Figure 5B). Use of commercial media, including HepatoSTIM, keratinocyte growth media (KGM), and bronchial epithelial growth medium (BGM), are becoming more common, although no salivary gland-specific media exists to date. There was significant overlap between different categories (ex. serum-containing media that was also supplemented with growth factors) as indicated by the Venn diagram in Figure 5A, suggesting that a variety of soluble cues are used to promote salivary gland cell growth, but specific studies contrasting individual benefits are lacking. Serum-containing media was more heavily used for culturing cell lines (Figure 5C), while Vitamin C (ascorbic acid) and transferrin were the main components of the media used for embryonic cells (Figure 5D), suggesting that cells may require a different optimized medium depending on cell source and cell maturity. A summary of some of the more promising soluble factors are provided in Table 2 and discussed further in the following sections.

#### 3.3.1. Growth Factors

Growth factors are important in salivary gland development, with FGFs contributing to branching morphogenesis and end bud formation [58,59], while EGF contributes to cell proliferation and differentiation along the ductal lineage [60]. EGF was added to the media in nearly all the studies using primary tissue, along with FGF2 in 25% of studies (Figure 5C). Other growth factors that are important to salivary gland development but are less commonly used include FGF7, which is reported to promote end bud formation, and FGF10, which enhances duct elongation [59]. Similarly, Miyajima et al., 2011 reported FGF7-induced bud expansion and FGF10-induced duct elongation, but no effect of EGF was observed on branching morphogenesis [24]. Additionally, FGF10 has been shown to increase Mist1, Aqp5, α-amylase, and α-SMA while decreasing K5 [49]. Insulin-like growth factor-1 (IGF-1) has been shown to support growth and maintenance of the paracellular barrier function (tight junction localization, TER, dextran permeability) at levels similar to supplementation with FBS, indicating that IGF-1 could be used to replace some of the functions of FBS in a serum-free media [52].

#### 3.3.2. Chemical Inhibitors

##### ROCK Inhibitor

Rho-kinases (ROCKs) mediate important processes such as proliferation, motility, secretion, and cell shape [61]. In the salivary gland specifically, ROCKs play an important role in cleft formation and basement membrane positioning in epithelial tissue polarity [62,63]. In cell culture, ROCK inhibitors have been used to prevent dissociation-induced apoptosis and preserve stem cell populations [64,65] and thus have been used to promote cell survival in vitro in many different tissue types [64,66,67,68]. Additionally, it has also been shown that ROCK activation can lead to the formation of acinar-to-ductal metaplasia under chronic pancreatitis conditions [67], suggesting that ROCK inhibition may prevent undesirable cell plasticity.

ROCK inhibitor Y-27632 has been shown to enhance cell growth, survival, proliferation and amylase and c-Met expression in salivary gland cultures [53] as well as increase sphere forming percent of CD24^hi^/CD29^hi^ cells [35]. However, the effect of ROCK inhibitor treatment may be dependent on other factors present in the culture media. Koslow et al. 2019 found an increase in sphere number, size, and proliferation and a decrease in cleaved caspase 3 in serum-free media; however, the increase in sphere size was not apparent in serum-containing media [69]. Additionally, it may promote different cell populations—Y-27632 in serum-free media increased Kit+ cells, while K5+ cells were increased in serum-containing media [69], suggesting it may promote different cell phenotypes altogether. Y-27632 has been used in culturing several other tissues (pancreas, prostate, lacrimal gland) to decrease cell stress [66,67,68], which may account for the increased growth in ROCK inhibitor-treated cultures. Conversely, it has also been shown to prevent salisphere formation [25] and cause cells to spread out and adhere to the surface instead of forming aggregates [25,53]. This may be a drawback to using Y-27632, since the increase in adhesion likely leads to a loss of 3-dimensional structure, organization, and, consequently, the secretory function of the salivary gland cells.

##### EGFR Inhibitors

The epidermal growth factor receptor (EGFR) modulates various processes, including proliferation, differentiation, and survival [70], and is involved in branching morphogenesis during salivary gland development [71]. However, aberrant EGFR signaling can lead to cancer and other diseases [70] and EGFR inhibition has been used as treatment for breast, lung, and colorectal cancer [67,68,69].

Several EGFR inhibitors have been used in salivary gland culture, including AG1478, PD198509, PD168393, and EKI-785. AG1478 retained epithelial cells and AQP5 [22], while PD198509 did not affect K5, K19, Kit, sphere diameter or sphere count [23] and PD168393 inhibited carbachol (CCh)-mediated morphogenesis and proliferation of K5+ and K19+ cells [60]. This suggests that EGFR inhibitors may be beneficial in promoting acinar cells/AQP5 expression, but not keratin-expressing duct cells. This is supported by the role of EGFR signaling in duct morphogenesis and differentiation and proliferation of the duct lineage [72] and by pancreas literature in which EKI-785 was used to prevent the transition of acinar cells into duct-like clusters in vitro [73]. This also highlights a key consideration in the selection of an optimized media—different goals may warrant different soluble cues. For example, researchers interested in isolating K5+ or Kit+ cells may benefit from having ROCK inhibitor in their media, but not EGFR inhibitor.

##### TGFβR Inhibitors

TGFβ has multiple roles in the salivary gland—it is important during morphogenesis [74], but it is also upregulated following stress, a major driver of fibrosis [75] and overexpression of TGF-β1 can lead to acinar loss [72,76]. Hence, it has been studied in salivary gland culture with different outcomes. The TGFβR inhibitor, RepSox, promoted cell growth, proliferation, expression of keratins 8, 14, and 19 and was selective for p63-expressing cells [77], while treatment with SB525334 increased acinar characteristics [50]. Since both inhibitors target TGFβR1, differences between these outcomes could be due to inhibitor potency, differences in culture conditions (tissue culture plate with commercial CnT-PR media [77] vs. growth factor-reduced Matrigel with DMEM/N2 media [50]), or difference in cell source (embryonic [77] vs. adult [50]).

#### 3.3.3. Neurotrophic Factors

The salivary gland is highly innervated, and salivation is controlled by the autonomic nervous system [78]. Hence, adding neurotrophic factors to the culture media has been widely considered as an alternative to the complexity of co-culturing salivary gland cells with nerve cells. Results show that treatment with different nerve factors has positive effects on salivary gland cultures. Neurotrophin 4 (NT-4) increased levels of amylase [54], neurturin (NRTN) initiated branching and innervation [23], and neureglin 1 (NRG1) promoted branching and retention of acinar-like cells [79]. This is supported by a myriad of publications highlighting the importance of neurotrophic factors such as neurturin and glial cell-derived neurotrophic factor (GDNF) for development of the salivary gland [60,78,80,81,82,83].

#### 3.3.4. Conditioned Media

Conditioned media from a variety of sources has also been shown to improve salivary gland cell culture. Wnt and R-spondin conditioned media increased long-term expansion of salivary gland stem cells in vitro, with increased population doubling and sphere-forming efficiency [55]. Mesenchymal stem cell (MSC)-conditioned media increased acinar-like structures and Aqp5 and K14 when combined with laminin-111 [56]. Fibroblast-conditioned media increased amylase protein levels [84] and amylase expression [54] but was dependent on the substrate the fibroblasts were grown on [54].

#### 3.3.5. Soluble ECM Proteins

In addition to variations in the biomaterials used for the matrix, soluble ECM proteins have also been used to provide signaling cues to the salivary gland cells as media additives. For example, fibronectin induced branching and ductal elongation [85], which was enhanced with FGF7. Salivary gland ECM extract (s-Ecx) promoted a compact sphere structure and increased expression of keratins (K5, K7, K14, K19) and acinar markers such as Aqp5 and Muc-1 [86]. Chitosan has been shown to increase spheroid size and polarization [25], with the greatest effect from soluble chitosan.

## 4. Opportunities for Future Research

Some of the most promising approaches for salivary gland cell culture involve the combination of the tissue engineering triad—cells, matrices, and soluble cues. While certain substrates have been shown to promote growth and expression of acinar markers, matrices are more relevant to the in vivo environment and versatile, providing structure, signaling through integrins, allowing for entrapment of signaling molecules and modifications with matrix motifs. In particular, laminin-based biomaterials have shown promising results [22,23,35,55,79]; this is supported by the prevalence of laminin in the ECM and basement membrane of the salivary gland in vivo [41].

Despite the promising polarization supported by matrix mimetics, secretory function remains limited. This continued challenge points to the need for incorporating combinatory approaches that optimize the matrix along with the other arms of the tissue engineering paradigm. While a number of groups have tested one or two matrix and/or media conditions, a large scale, combined media, and matrix optimization has not been done. In addition, analysis of how these factors affect a wide variety of markers, both acinar and duct, would be beneficial for more widespread adoption across the field.

Increasing complexity of models by introducing mesenchyme and neurons will enable more representative tissue mimetics for fundamental biology studies. However, simplicity may be desired in other cases for increased convenience, lower costs, and ease of data interpretation. Thus, it would be beneficial to investigate the specific function provided by supportive tissues and whether media supplementation or matrix modification can produce the same results.

## 5. Conclusions

Traditional salivary gland tissue engineering approaches fail to provide the necessary conditions to promote secretory function. Researchers have addressed this issue by co-culturing with neural or mesenchymal tissue, investigating different biomaterials and supplementing culture media with a variety of soluble cues. A key consideration in determining the optimal conditions depends on the goal of the work, as some seek to increase acinar cell characteristics, while others are concerned with increasing expression of putative stem cell markers or mimicking branching morphogenesis. Specific soluble factors that have increased the acinar phenotype include FGF10, neurotrophic factors, and EGFR inhibitors. The use of matrix mimetics, such as Matrigel^®^, collagen, and functionalized synthetic polymers, rather than just inert substrates, provide increased opportunities to improve the acinar cell phenotype. Factors that may improve stem cell maintenance include ROCK inhibitors and Wnt/R-spondin conditioned media, with mixed results from TGFβR1 inhibitors and MSC-conditioned media.

Based on the current state of the field, this study has revealed there is room for further optimization to recapitulate the in vivo salivary gland using in vitro culture models. Studies are needed to simultaneously optimize the combination of soluble factors with matrix cues and it is intriguing to consider improvements that maybe gained by co-culturing salivary glands cells with supportive tissues, such as neural or mesenchymal tissue as described by Vining et al., 2019 and Hosseini et al., 2018. Other pioneering approaches could include the use of microfluidic devices or 3D printing technology to aid in increasing the complexity and relevance of cell culture models, such as those developed for other tissues [87,88,89,90,91].

## Figures and Tables

**Table 1:** Categorization of information extracted from selected articles.

Cell Source	Cell Maturity	Cell Species	Gland Type	Media Type	Substrate
Primary Cell line Both	Adult Embryonic Both	Human Mouse Rat Other Multiple	Submandibular Parotid Minor Other Multiple	Growth Factor Serum Commercial	Matrix mimetic Plastic Transfer Specialized Multiple Membrane Unreported

**Table 2:** Soluble factors and matrices for improving cultured cell function.

Soluble Factor	Cells	Substrate/Matrix	Outcomes	
Fibroblast growth factor 10 (FGF10)	Human submandibular	Matrigel^®^	Increased Mist1, AQP5, α-amylase and α-SMA, decreased K5, promoted budding with no effect on organoid size, increased carbachol-induced calcium release	[49]
Epidermal growth factor receptor (EGFR) inhibitor	Mouse E16 submandibular	Nuclepore filter	EGFR inhibitor AG1478 retained epithelial cells and AQP5	[22]
Transforming growth factor β receptor I (TGFβRI) inhibitor	Mouse submandibular	Matrigel^®^	Treatment with TGFβ and TGFβR-I inhibitor SB525334 increased expression of amylase-1, Aqp5, ZO-1, Occuldin, Fgf7 and Fgf10 but not collagen type I when cultured on Matrigel and increased the size of acinar clusters	[50]
Epiregulin	Mouse submandibular	Collagen-coated culture dish	Increased cell proliferation and levels of EGFR ligands epiregulin, HB-EGF, amphiregulin and TGFα	[51]
Insulin-like growth factor I (IGF-I)	SMIE cell line (rat submandibular)	Collagen-coated Transwell	Treatment with 100 ng/mL IGF-I maintained cell number, cell viability, tight junction expression and localization and paracellular barrier function	[52]
Rho-associated protein kinase (ROCK) inhibitor Y-27632	Mouse submandibular	Matrigel^®^	Enhanced growth (cell numbers), survival (Live/Dead), proliferation (EdU), motility (scratch assay), maintained α-amylase expression and induced C-Met expression	[53]
Neurotrophin 4 (NT-4)	Human parotid	Cell culture plate	Highest levels of intracellular and secreted α-amylase at 1 ng/mL NT-4	[54]
Neurturin (NRTN)	Mouse or human submandibular with E13 mesenchyme and neuronal cells	Laminin-111 hydrogel	Initiated branching, innervation and self-aggregation of spheres	[23]
Neureglin 1 (NRG1)	Human parotid	Cell culture plate	Promoted branching and retention of acinar-like cells in submandibular	[54]
Wnt and R-spondin conditioned media	Mouse submandibular	Matrigel^®^	Increased population doubling and sphere-forming efficiency	[55]
Mesenchymal stem cell (MSC) conditioned media	Mouse submandibular	Matrigel^®^ or laminin-1	Increased acinar-like structure and when combined with laminin-111, increased AQP5 and K14 and decreased in α-SMA compared to fresh submandibular gland	[56]
p38 MAPK inhibitor/Src inhibitor	Rat parotid	Collagen-coated dish or cell culture insert	Reduced cell stress	[57]

AQP5: aquaporin-5; α-SMA: α-smooth muscle actin; K5: keratin 5; K14: keratin 14; Nkcc1: sodium-potassium-chloride channel 1; ZO-1: zonula occludens 1; HB-EGF: heparin-binding EGF-like growth factor; TGF-α: transforming growth factor-α.

## Data Availability

The data presented in this study are available on request from the corresponding author.
